# Longitudinal Links Between Adolescent Social Anxiety and Depressive Symptoms: Testing the Mediational Effects of Cybervictimization

**DOI:** 10.1007/s10578-018-0829-1

**Published:** 2018-07-17

**Authors:** Nejra Van Zalk, Maarten Van Zalk

**Affiliations:** 1 Dyson School of Design Engineering, Imperial College, London, SW7 2AZ UK; 2 Developmental Psychology, Osnabrück University, Osnabrück, Germany

**Keywords:** Social anxiety, Depressive symptoms, Cybervictimization, Comorbidity, Early adolescence

## Abstract

This study focuses on the temporal sequence between social anxiety and depressive symptoms, and whether cybervictimization might mediate these links. We used a longitudinal sample of 501 early adolescents (51.9% girls; M_age_ = 13.96) followed at three time points. Using a cross-lagged path model in MPlus, we found that social anxiety predicted depressive symptoms over time, but not the other way around. Time-1 depressive symptoms also predicted cybervictimization, but only for boys and not for girls. No mediating effects of cybervictimization emerged; however, Time-2 social anxiety was a significant mediator between Time-1 social anxiety and depressive symptoms, whereas Time-2 depressive symptoms significantly mediated the link between Time-1 social anxiety and Time-3 depressive symptoms. In sum, social anxiety was a strong predictor of depressive symptoms over time but not vice versa—irrespective of cybervictimization.

## Introduction

**Fig. 1:**
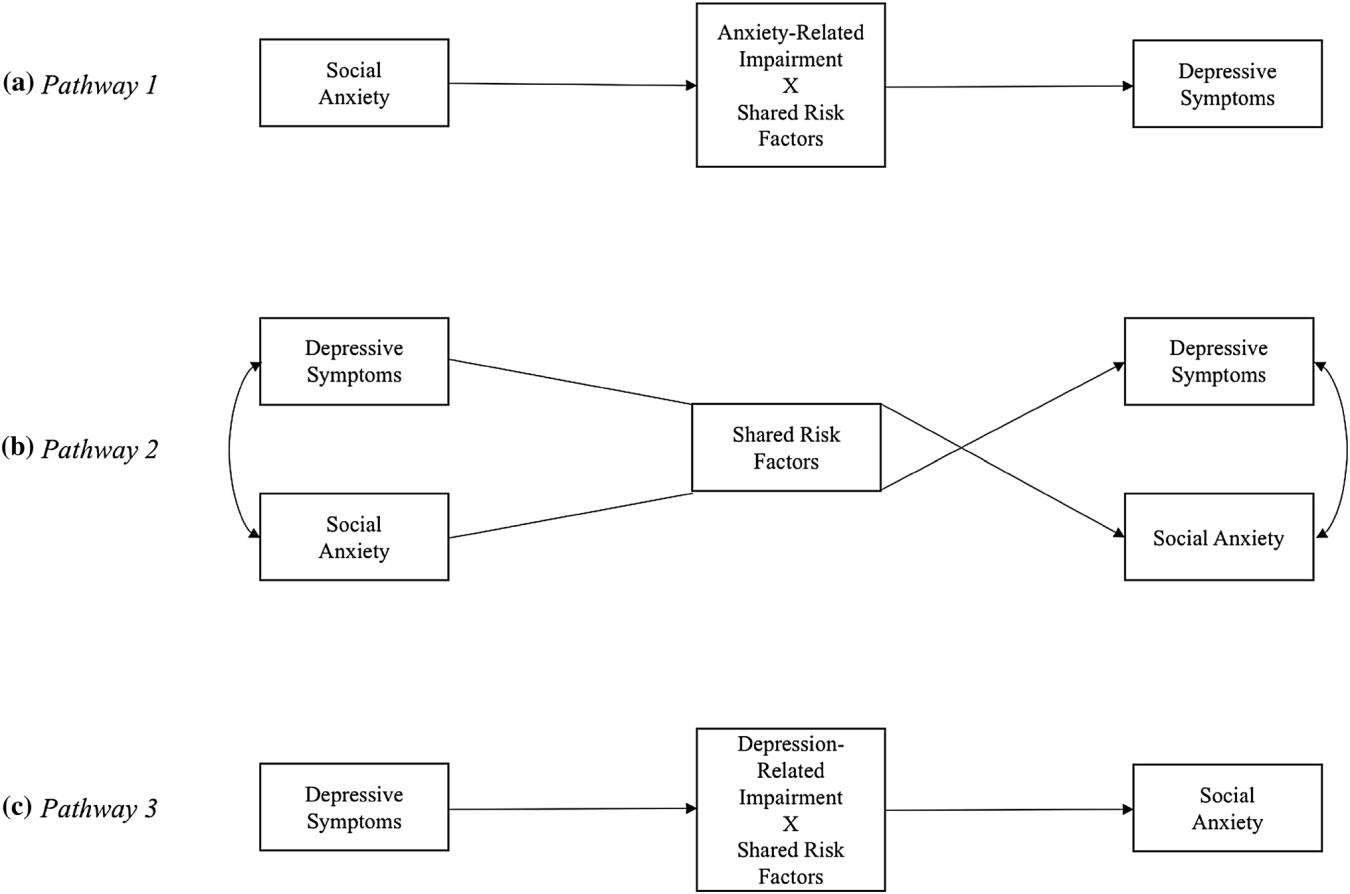
Proposed multiple pathways model (Cummings et al. [4]) depicting potential pathways between social anxiety and depressive symptoms over time

Early adolescence is considered a transitional phase during which young people are at risk for developing various internalizing problems. Specifically, early adolescence is typically the time of onset for both non-clinical social anxiety [1, 2] and depressive symptoms [3, 4], which tend to increase during this time period [5], and are likely forerunners for developing social anxiety disorder as well as major depressive disorder later on in life [6, 7]. The comorbidity between social anxiety and depressive symptoms is common in adolescence [2] and has received considerable attention in the literature. According to the recently proposed *multiple pathways* model (depicted in Fig. 1), this comorbidity might be explained via three possible pathways between social anxiety and depressive symptoms [4]. The *first* pathway (illustrated in the upper part of Fig. 1) refers to adolescents with a specific vulnerability for social anxiety only; which, if it develops and is left untreated, becomes a risk factor for developing subsequent depression due to anxiety-related impairment [4]. The *second* pathway (as shown in the middle of Fig. 1) refers to adolescents who develop symptoms of social anxiety and depression simultaneously because they already have a shared diathesis for both [4]. Finally, the *third* pathway (shown in the lower part of Fig. 1) involves adolescents with a primary vulnerability for depression only, who subsequently develop social anxiety due to depression-related impairment [4]. Understanding more about whether early adolescent social anxiety precedes depressive symptoms, whether depressive symptoms precede social anxiety, or whether there are bidirectional links between the two, is thus crucial in order to prevent increases in the development of these problems over time.

The majority of the current research indicates that social anxiety is a strong precursor for depressive symptoms, thus confirming the first pathway in the proposed multiple pathways model [4]. One explanation is that socially anxious adolescents’ social skills deficits likely increase the risk for being rejected, victimized, and excluded by peers [8]. By appearing awkward and socially inept, socially anxious adolescents tend to alienate peers, which likely leads to feelings of hopelessness, lower self-esteem, and poor friendship quality—consequently increasing depressive symptoms [9]. Socially anxious adolescents also tend to avoid social interactions, leading to a vicious circle of increased social seclusion and loneliness [8], which might increase depressive symptoms [10]. In addition, trajectories of social anxiety have been found to predict increases in depressive symptoms, but not vice versa [11]. Nevertheless, there is also evidence that depressive symptoms significantly contribute to the development of social anxiety due to interpersonal impairment linked with appearing depressed—supporting the third pathway in the proposed model [4]. Adolescents’ apparent depressed mood might prompt peer-related difficulties, such as for example excessive reassurance-seeking about social incidents [12], and they might start fearing social interactions and negative evaluations—which could contribute to the development of social anxiety over time [13]. Finally, the proposed second pathway in the multiple pathways model has been somewhat supported in the current literature. In one study, high levels of interpersonal stressors have been linked to higher levels of initial depressive symptoms as well as social anxiety, which in turn predicted higher levels of both over time [14]. Nevertheless, there are many conceptual and methodological differences between the compared studies, and most are cross-sectional—making it important to examine the three suggested developmental pathways within one single model with a longitudinal design.

Early adolescence represents a qualitative change in social dynamics that appears to create fertile ground for peer victimization [15], which is the interpersonal stressor of particular focus for the current study. There are several ways in which victimization in particular might be harmful in terms of co-morbidity between social anxiety and depressive symptoms. Research indicates that displaying symptoms of depression or anxiety toward peers might send a signal of weakness during a phase when many young people want to exercise authority against those who appear weaker—thus prompting early adolescents to victimize more depressed and anxious peers [15]. Indeed, peer-related difficulties are closely related to social anxiety [2], and social anxiety is a well-known risk factor for victimization [16]. In addition, victimization appears to be both a predictor and a consequence of social anxiety over time [17], and increases in boys’ social anxiety particularly have been associated with increases in relational victimization [13]. In a longitudinal study evaluating the pathways in the abovementioned multiple pathways model, the authors examined peer victimization such as social exclusion as a mediator between social anxiety and depressive symptoms [14]. Peer victimization during the previous year was related to social anxiety, whereas social anxiety subsequently predicted higher levels of social anxiety 2 years later [14]. Links between depressive symptoms and peer victimization have also been shown in the literature. For example, internalizing problems such as depression have been linked to being victimized in a recent review [18]. Thus, peer victimization is an important interpersonal stressor associated with both social anxiety as well as depressive symptoms in early adolescence, perhaps especially so for boys.

The majority of the current literature on the links between internalizing and victimization has focused on offline victimization, however. Nevertheless, online victimization, or *cybervictimization*, can be more difficult to escape compared to for example being bullied in school [19]. Studies on cybervictimization show that it is linked to emotional difficulties regardless of offline victimization [19–22]. Several literature reviews have revealed positive correlations between cybervictimization and depressive symptoms [23, 24], as well as between cybervictimization and social anxiety [25]. In one short-term longitudinal study (6 weeks between the time points) looking at victimization both online and offline and controlling for comorbidity between social anxiety and depressive symptoms, the results indicated that cybervictimization was linked to increased depressive symptoms, whereas offline victimization was associated with increased social anxiety over time [25]. The authors concluded that cybervictimization might be uniquely linked to depressive symptoms rather than social anxiety and should receive significant focus in preventive interventions for depression [25]. In another study, however, social anxiety but not depressive symptoms showed weak cross-sectional links (*β* = 0.15) to cybervictimization [26]. Thus, there is inconsistent evidence in the literature, and the only longitudinal study indicates that cybervictimization predicts depression rather than social anxiety. To our knowledge, however, no studies have yet investigated the mediating role of cybervictimization on the links between social anxiety and depressive symptoms over time.

Finally, gender differences in all of these processes might be expected for several reasons. First, girls tend to have higher mean levels of both social anxiety as well as depressive symptoms compared to boys [4]. Despite mean-level differences, however, being socially fearful might be more accepted for girls than boys [27]. In addition, it appears that girls’ social fears are more malleable, as their social anxiety is more strongly influenced by their peers’ levels of social anxiety compared to boys’ social anxiety [28], which might be particularly important in the context of being victimized by online peers. Nevertheless, a previous direct test of the multiple pathways model did not identify gender differences on these processes [14]. In terms of cybervictimization, studies indicate that girls might be more cybervictimized compared to boys [29], whereas boys who are cybervictimized report slightly higher levels of somatic symptoms compared to girls [19]. Thus, gender could both be a confounding variable as well as a moderator for all of the aforementioned processes.

In this study, we aim to study the links between social anxiety and depressive symptoms over time in a sample of early adolescents followed up at three time points (with approximately 8 months between the time lags), and test for indirect effects of cybervictimization. We consider the use of three measurements over time with depressive symptoms, social anxiety, and cybervictimization in one model a distinct advantage of this study compared to prior research, because a full mediation model and alternative mediation models (e.g., cybervictimization effects on social anxiety being mediated by depressive symptoms) can be examined with a three-wave design [e.g., 30, 31]. We aim to empirically test the multiple pathways model and extend it by examining the mediating effects of cybervictimization as a potential interpersonal stressor in early adolescence. By using all three variables together in one model with a longitudinal design, all the proposed pathways in the multiple pathways model can be examined simultaneously. We use adolescent self-ratings of social anxiety, depressive symptoms, and cybervictimization. Based on the previous literature showing that social anxiety usually precedes depressive symptoms [4], we hypothesized that social anxiety would predict cybervictimization, which would in turn predict depressive symptoms over time (thus supporting pathway 1 in the proposed model). As there have been studies showing that depressive symptoms also precede social anxiety [4], we hypothesized that depressive symptoms would predict social anxiety and that this link would be mediated by cybervictimization (supporting pathway 3). Because there is less support for the second pathway in the proposed multiple pathways model [4], we hypothesized that cybervictimization would not contribute to the simultaneous development of social anxiety and depressive symptoms. Finally, though there might be important gender differences in the bidirectional links between social anxiety, depressive symptoms, and cybervictimization, prior results have found mixed results. Nevertheless, past studies have not included all three variables in a single model to examine gender differences. Given the previously mentioned disparities in findings regarding gender, therefore, we did not develop a priori gender hypotheses, but rather explored the moderating effects of gender for all of the above mentioned processes.

## Method

### Participants

The data are from a longitudinal project focusing on the role of online and offline friendships in early adolescent emotional adjustment. Participants were early adolescents (7th–9th —graders) from a medium-sized town in Sweden with a population of about 130,000. The data collections took place in school and online with approximately 8 months between the time lags. The first data collection took place in September 2010 (Time 1), followed by the second measurement in May 2011 (Time 2) and a final measurement in January 2012 (Time 3). The initial sample included 423 adolescents 7th–9th graders from one school (205 girls; *M*_*age*_ = 14.05). There were three classrooms per grade, across which the participants were evenly distributed. At the onset of the study, about 12.1% of all participants were ethnic minorities, which was slightly lower compared to 14.7% in the entire country, according to official reports [32]. Mean incomes were about 5% lower, whereas the unemployment rates (6%) and the proportion of single-parent households in the community (5.1%) were similar to the rest of the country [32].

Measures were collected via a combination of in-school offline surveys collected at Times 1 and 2, and online surveys collected at Times 1–3. During the Time-1 offline surveys, the adolescents who were initially recruited provided their e-mail address. They were subsequently sent a link with a specified username and password to complete the online part of the survey. During the Time-1 online survey, the adolescents nominated close friends who were in turn sent an e-mail invitation to the study if they had not already participated. The procedure for the friends’ data collection mirrored the data collection of the targets (including own and parental consent), with the exception that all questionnaires were filled out online. This added another 312 participants, of which 51 provided mostly no data (as they stopped filling out the online questionnaires almost directly after starting) and were thus removed. This resulted in a sample of 683 participants.

In order to be included in the analytic sample for the current study, the participants had to be early adolescents with at least two waves of data available. Youths younger than 13 (9–12 in the final sample) and older than 15 (16–31 in the final sample) had high levels of data missingness for the study variables (43–84%), and the majority of them did not participate in more than one wave of the study. They were therefore removed, and the final target sample for this study thus comprised 501 13–15 year-olds (51.9% girls; *M*_*age*_ = 13.96). *t*-tests revealed no significant mean differences between the 501 participants and those excluded from the final sample (*n* = 182) on any of the measures in the current study.

To calculate an estimated proportion of all available observations for each variable in the analyses (or the proportion of missing values in the data set), we used the covariance coverage matrix in MPlus [33]. The participants in the final analytic sample had between 51–82% of data available for the individual measures over time. The missingness in the data is associated with the nature of the data collection itself. Because the data collection was mainly focused on 13–15 year-olds attending lower secondary school, the participants who left the school when they were 15 to attend higher secondary school have data missing at each subsequent time point. Nevertheless, Little’s MCAR test showed that the data were missing completely at random (*sig*. = 0.41).

## Procedure

The adolescents provided consent before filling out the offline questionnaires when they were visited by trained research assistants in their classrooms during school time. The teachers were not present. The adolescents were told about the types of questions they would answer, and the time it would take to finish the questionnaires. They were also informed that their participation was voluntary, that they could do something else, and that their answers would never be shown to anyone if they chose to participate. The participants filled out the online questionnaires during the time of their choosing following the offline data collection. Prior to the start of the data collection, the parents were informed about the study through a meeting at the school. They were subsequently sent a pre-paid post card along with additional information about the study to return if they did not want their child to participate in the study (only 2% of the parents did so). They were informed that they could withdraw their child from the study at any time. No participant was paid for taking part in the study, but the adolescents received two gift cards for cinema tickets—whether or not they chose to participate. All the procedures and measures used in this study were approved by the Regional Ethics Committee.

## Measures

### Social Anxiety

We measured non-clinical social anxiety with questions about fears in different social situations [34]. This instrument is a modified version of the Social Phobia Screening Questionnaire, which was originally created for adults [35] and adjusted for children and adolescents up to age 18 (the SPSQ-C, or the Social Phobia Screening Questionnaire for Children; 34). In a validation study, this instrument has showed a moderate test–retest reliability, *r* = .60 [36].The measure comprises eight questions about how much fears the adolescents felt in various social situations, such as “being with classmates during breaks.” The response items ranged from *None* (1), *Some* (2), to *A lot* (3). The Cronbach’s *α*’s were 0.72 for Time 1, 78 for Times 2 and 3.

### Depressive Symptoms

We measured depressive symptoms using a shortened version of the Child Depression Scale from the Center for Epidemiological Studies (the CESD-10; [37]). This scale assesses depressive symptoms such as worry, sadness, hopelessness, lethargy, and poor appetite, and has demonstrated good factorial validity [38]. The shortened version measures non-clinical symptoms and includes ten questions based on a factor analysis conducted on the original 20-item scale. Participants were instructed to think about the past week, with questions such as “How often have you worried about things you don’t usually worry about?” The response items ranged from *Not at all* (1), *Occasionally* (2), *From time to time* (3), to *Often* (4). The Cronbach’s *α*’s were 0.82 for Time 1, 0.86 for Time 2, and 0.88 for Time 3.

### Cybervictimization

We asked nine questions about being bullied while communicating with others online [39]. The original scale was based on the Olweus Bully/Victim Questionnaire [40], but adapted to focus on aggressive acts in chat situations, so as to assess being victimized in online social contexts. This measure has shown good internal consistency and empirical distinctiveness from offline victimization in a validation study [39]. At the time of the data collection (from 2010 to 2012), a Swedish report concluded that chatting to friends using Instant Messaging or chatrooms was by far the most common use of online communication by early adolescents during the time of the data collection [41].

Before reading the cybervictimization items, the participants in our study received the following instructions about what we meant by chatting: “*These questions are about ´chatting´. By chatting we mean being online and sending messages directly to other people who can answer you straight away (like using MSN, for example). Chatting can be done via your computer or your mobile phone. Chatting is NOT leaving messages on forums, blogs, e-mailing, texting, or any other way of communication that does not involve immediate contact.”* An item example was “How often are you harassed for no apparent reason?” The response items ranged from *Never* (1), *Only once or twice* (2), *2 or 3 times a month* (3), *More than once a week* (4), to *Daily* (5). The Cronbach’s *α*’s were 0.86 for Time 1, 0.83 for Time 2, and 0.88 for Time 3.

To further test for construct validity of the cybervictimization measure, we correlated it with the widely used Rosenberg self-esteem scale [42] across the three time points. Recent meta-analyses of the current literature have shown a negative association (*r* = − .22) between self-esteem and peer [43] as well as cybervictimization (*r* = − .17; 44). In our study, correlations between self-esteem and cybervictimization ranged from − 0.13 to − 0.33 across the three time points (all *p*’s < 0.05). As the cybervictimization measure was related to self-esteem according to previous theoretical expectations and findings from meta analyses, this demonstrated construct validity.

### Length and Frequency of Chatting

Two items were used to measure how long and how often adolescents chatted with others online. The first question was “How often do you chat per week?” The response items ranged between *None of the days* (1), *1 day*/*week* (2), *2 days*/*week* (3), *3 days*/*week* (4), *4 days*/*week* (5), *5 days*/*week* (6), *6 days*/*week* (7), to *All days of the week* (8). The second question was “If you chat, how long do you chat each time?” The response items ranged from *Never* (1), *Up to one hour* (2), *1–2 h* (3), *2–3 h* (4), *3–4 h* (5), *4–5 h* (6), *5–6 h* (7), to *More than 6 h* (8). The correlations between the two items were 0.48 for Time 1, 0.54 for Time 2, and 0.50 for Time 3.

## Strategy of Analysis

In order to assess the links between all study variables, cross-lagged path models with manifest variables were conducted using MPlus 7.0 [33] and the FIML (Full Information Maximum Likelihood) procedure. FIML makes use of all available data to estimate information about missing data in the dataset, thus producing unbiased parameter estimates when data are missing at random [30, 45]. As such, FIML provides less biased results than both pairwise and listwise deletion by estimating data [30].

As a first step, we examined the links between social anxiety, cybervictimization and depressive symptoms across the three time points. For model fit indices, we used the root mean square error of approximation or RMSEA (with values between 0.05 and 0.08 considered an acceptable fit), as well as the comparative fit index or CFI (where values between 0.90 and 0.99 are considered acceptable fit; 30). We included the following covariations and paths in the model: (a) within-time covariations between all variables at Time 1–2 and Time 2–3, (c) stability paths for all variables from Time 1–2 and Time 2–3, and (d) directional cross-lagged paths between all variables from Time 1–2 and Time 2–3.

Boys and girls were compared by means of multiple-group comparison procedures, thus examining moderation effects. The gender groups were tested for significant differences on the paths of theoretical interest in the model. That is, only the directional paths (i.e., the stabilities and the cross-lagged paths) were included in the group testing. The within-time variances were not included as they were not of theoretical interest and were hence left constrained throughout the testing procedure. First, all of the parameters in the model were constrained to be equal between the two groups. *χ*^*2*^—difference tests were then conducted by releasing each path of interest in the model one by one, thus guiding the retention of the paths ultimately used in the final, best-fitting model (determined by significant increases in model fit indices). Paths in the model that were significantly different between the two groups were interpreted as moderating effects of gender. All paths presented in the results are standardized.

In order to test for mediating effects of cybervictimization on the links between social anxiety and depressive symptoms, indirect effects comparing boys and girls were tested via the Monte Carlo method (as outlined in 31) with bootstrapping in MPlus [33]. For all indirect effects, we attained bias-corrected confidence intervals of indirect effects using 5000 resamples [46]. To evaluate the significance of the indirect effects, MPlus produces 95% asymmetric confidence intervals (considered statistically significant if the 95% CI did not contain zero).

## Results

### Descriptives

The descriptives for all study variables are shown in Table 1, and the correlations are shown in Table 2. As the table shows, depressive symptoms and social anxiety were significantly correlated across all time points. Depressive symptoms were also correlated with cybervictimization across nearly all time points. Time-1 social anxiety was related to Time-1 cybervictimization, whereas Time-3 social anxiety was related to cybervictimization at Times 2 and 3. In addition, girls had higher levels of social anxiety and depressive symptoms compared with boys, as would be expected—as girls tend to have higher mean levels of non-clinical social anxiety and depressive symptoms compared to boys [4]. There were no significant mean gender differences on cybervictimization, however.

**Table 1:** Raw-score means (standard deviations) with *t*-tests for differences between girls and boys

	Total	Girls	Boys	Statistic
Social anxiety T1, *M* (*SD*)	1.38 (0.30)	1.49 (0.30)	1.27 (0.26)	*t* (393) = 7.56***
Social anxiety T2, *M* (*SD*)	1.37 (0.33)	1.48 (0.33)	1.25 (0.30)	*t* (373) = 7.23***
Social anxiety T3, *M* (*SD*)	1.47 (0.40)	1.56 (0.41)	1.34 (0.35)	*t* (255) = 4.51***
Cybervictimization T1, *M* (*SD*)	1.33 (0.56)	1.30 (0.52)	1.37 (0.60)	*t* (362) = − 1.18
Cybervictimization T2, *M* (*SD*)	1.24 (0.46)	1.22 (0.37)	1.27 (0.57)	*t* (277) = − 1.012
Cybervictimization T3, *M* (*SD*)	1.25 (0.50)	1.22 (0.49)	1.29 (0.52)	*t* (265) = − 1.02
Depressive symptoms T1, *M* (*SD*)	1.86 (0.58)	1.99 (0.60)	1.73 (0.52)	*t* (392) = 4.74***
Depressive symptoms T2, *M* (*SD*)	1.84 (0.62)	2.05 (0.67)	1.62 (0.49)	*t* (407) = 7.42***
Depressive symptoms T3, *M* (*SD*)	2.00 (0.68)	2.14 (0.69)	1.78 (0.61)	*t* (256) = 4.18***
Frequency of chatting T1, *M* (*SD*)	5.67 (0.13)	5.64 (2.40)	5.68 (2.49)	*t* (333) = − 1.15
Frequency of chatting T2, *M* (*SD*)	5.28 (0.15)	5.42 (2.37)	5.09 (2.75)	*t* (277) = 1.05
Frequency of chatting T3, *M* (*SD*)	4.81 (0.16)	4.74 /2.63	4.90 (2.67)	*t* (267) = − 0.49
Length of chatting T1, *M* (*SD*)	3.56 (0.09)	3.46 (1.62)	3.68 (1.82)	*t* (333) = − 1.15
Length of chatting T2, *M* (*SD*)	3.33 (0.11)	3.42 (1.75)	3.21 (1.58)	*t* (249) = 0.98
Length of chatting T3, *M* (*SD*)	3.07 (0.10)	3.09 (1.51)	3.05 (1.48)	*t* (227) = 0.17

Note: *p < .05, **p < .01, ***p < .001

**Table 2:** Correlations between main study variables

	1.		2.		3.		4.		5.		6.		7.		8.	
1. Social anxiety T1	–															
2. Social anxiety T2	0.73	***	–													
3. Social anxiety T3	0.61	***	0.64	***	–											
4. Cybervictimization T1	0.13	*	0.11		0.003											
5. Cybervictimization T2	0.10		0.11		0.16	*	0.54	***	–							
6. Cybervictimization T3	0.04		0.01		0.27	***	0.31	***	0.32	***	–					
7. Depressive symptoms T1	0.23	***	0.24	***	0.17	*	0.36	***	30	***	0.16	*	–			
8. Depressive symptoms T2	0.25	***	0.29	***	0.24	***	0.22	***	.29	***	0.07		0.66	***		
9. Depressive symptoms T3	0.37	***	0.34	***	0.48	***	0.15	*	0.34	***	0.31	***	0.52	***	0.56	***

Note: *p < .05, **p < .01, ***p < .001

As can be seen in Table 1, even though there were no significant differences between boys and girls on frequency or length of chatting, the participants reported chatting nearly at least 4 days a week, and between 2 and 3 h at the time on average across the three time points. 92, 90, and 85% of the youths reported chatting at least 1 day a week, with 38, 33, and 29% of the adolescents chatting online every day at Times 1, 2, and 3, respectively. Only 0.2% of the adolescents reported never chatting (and only at Time 1), 21.1–23.2% chatted up to one hour, 9.3–14.6% chatted 1–2 h, 5.7–9.9% chatted 2–3 h, 3.2–7% chatted 3–4 h, 1.5–4.4% chatted 5–6 h, and 1.7–3.2% chatted for more than 6 h at a time across the three time points. These numbers are similar to another Swedish report published at the time of the current data collection [41], and reflect the fact that early adolescents spent a lot of time chatting online—potentially providing ample opportunities for being victimized in online settings.

### Testing the Longitudinal Links Between Social Anxiety, Depressive Symptoms and Cybervictimization and the Moderating Effects of Gender

To test our main hypotheses, we conducted a cross-lagged path model with social anxiety, depressive symptoms, and cybervictimization as main variables. We included within-time co-variation paths between all variables at all time points, as well as stabilities and directional paths from Time 1 to Time 2, and from Time 2 to Time 3 for all variables. This baseline model had an adequate fit (*χ*^2^ = 43.82; *df* = 10; *p* < .0001; RMSEA = 0.08; CFI = 0.96), and the results are shown in the first column of Table 3. The within-time co-variation paths between all variables ranged between 0.06 (*n.s*.) and 0.40 (*p* < .001). As can be seen in Table 3, social anxiety predicted depressive symptoms from Time 1 to Time 2, and from Time 2 to Time 3, but not the other way around. The relative magnitude of these paths was compared in order to determine whether they differed significantly. Comparing the unstandardized path coefficients in MPlus, the bias-corrected bootstrap confidence interval for the difference between parameters indicated that they were significantly different at both time points, 95% *CI*’s for Time 1–Time 2 [0.01, 0.53] and Time 2–Time 3 [0.10, 0.93]. Hence, social anxiety was significantly different in predicting depressive symptoms from depressive symptoms predicting social anxiety over time. Social anxiety also showed the highest stability over time, followed by cybervictimization and depressive symptoms. Jointly, these results support pathway 1 (but not pathway 3) in the proposed multiple pathways model (shown in Fig. 1).

**Table 3:** Standardized results for final model with χ^2^—difference tests for cross-lagged directional paths comparing girls and boys

Directional paths	β Baseline model		β Girls		β Boys		χ^2^ Difference test
Predicting T3 social anxiety							
Social anxiety T2	0.69	***	0.66	***	0.67	***	n.s.
Cybervictimization T2	− 0.01		0.01		0.01		n.s.
Depressive symptoms T2	− 0.04		− 0.06		− 0.05		n.s.
Predicting T2 social anxiety							
Social anxiety T1	0.72	***	0.72	***	0.67	***	n.s.
Cybervictimization T1	− 0.03		− 0.01		− 0.01		n.s.
Depressive symptoms T1	0.06		0.02		0.04		n.s.
Predicting T3 cybervictimization							
Social anxiety T2	− 0.03		− 0.01		− 0.01		n.s.
Cybervictimization T2	0.37	***	0.31	*	0.42	***	n.s.
Depressive symptoms T2	− 0.04		− 0.03		− 0.02		n.s.
Predicting T2 cybervictimization							
Social anxiety T1	0.02		0.04		0.03		n.s.
Cybervictimization T1	0.52	***	0.54	***	0.43	***	n.s.
Depressive symptoms T1	0.15	**	0.11		0.25	**	< 0.05
Predicting T3 depressive symptoms							
Social anxiety T2	0.23	***	0.21	**	0.23	**	n.s.
Cybervictimization T2	0.08		0.07		0.12		n.s.
Depressive symptoms T2	0.44	***	0.45	***	0.38	***	n.s.
Predicting T2 depressive symptoms							
Social anxiety T1	0.14	***	0.09	*	0.10	*	n.s.
Cybervictimization T1	− 0.01		0.02		0.03		n.s.
Depressive symptoms T1	0.64	***	0.58	***	0.72	***	n.s.

*n.s*. non-significant χ^2−^ difference between girls and boys

Note: *p < .05, **p < .01, ***p < .001

**Fig. 2:**
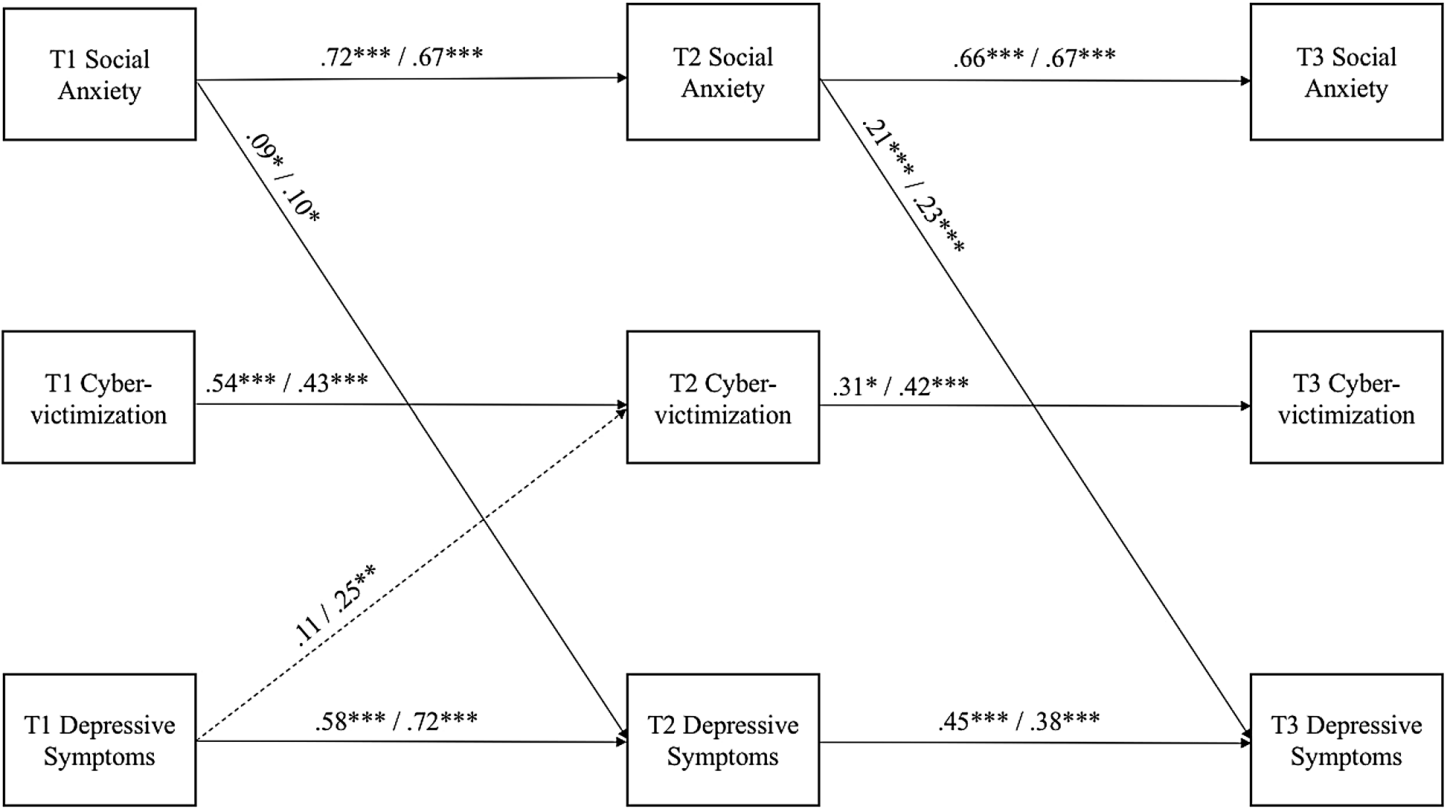
Cross-lagged path models with moderating effects of gender. Note. Left-hand values are for girls and right-hand values are for boys. For clarity, only significant directional paths are depicted in the figure, whereas paths with larger dotted lines represent significant group differences (according to χ^2^—difference tests). *p < .05, **p < .01, ***p < .001

As a next step, we tested for indirect effects of cybervictimization on the links between social anxiety and depressive symptoms, as well as the reverse (the link between depressive symptoms and social anxiety) from Times 1 through 3. We also examined how Time 1 variables predicted Time 3 variables via Time 2 variables (such as social anxiety and depressive symptoms indirectly affecting social anxiety and depressive symptoms over time, respectively), in order to test alternative mediation effects. One possibility is that initial depressive symptoms at Time 1 predict changes in social anxiety at Time 3 through subsequent depressive symptoms at Time 2. Furthermore, initial social anxiety at Time 1 might predict changes in depressive symptoms at Time 3 through subsequent social anxiety at Time 2. This is consistent with seminal literature on testing longitudinal mediation models in structural equational modelling [see e.g., 30, 47]. Simultaneously, we tested for the moderating role of gender for all the indirect effects. First, we constrained all of the parameters in the model to be equal between genders. *χ*^2^—difference tests were then conducted by releasing each path of interest in the model one by one, thus guiding the retention of paths ultimately used in the final, best-fitting model (determined by model fit indices). This final model had a good fit (*χ*^2^ = 75.90; *df* = 44; *p* < .001; RMSEA = 0.05; CFI = 0.96). The results for this model are shown in Table 3 and depicted in Fig. 2.

As can be discerned from the results, one significant gender difference emerged. Namely, Time-1 depressive symptoms predicted Time-2 cybervictimization for boys but not for girls. Regarding the indirect effects, cybervictimization was not a significant mediator between social anxiety and depressive symptoms, nor vice versa. Rather, Time-2 depressive symptoms were a significant mediator between Time-1 social anxiety and Time-3 depressive symptoms for both girls and boys (*Two-Tailed Estimate* = 0.02, *CI*_95%_ = 0.01–0.18, *p* < .05). Furthermore, Time-2 social anxiety was a significant mediator between Time-1 social anxiety and Time-3 depressive symptoms for both boys and girls (*Two-Tailed Estimate* = 0.38, *CI*_95%_= 0.18-0.59, *p* < .01). In sum then, social anxiety was a predictor of depressive symptoms but not vice versa, but cybervictimization did not mediate these links in the current study.

## Discussion

Using the framework of the recently proposed multiple pathways model [4], this study investigated the temporal sequence of early adolescent social anxiety and depressive symptoms, whether interpersonal stressors such as cybervictimization mediated these links, and whether there were differences for girls and boys. Our results show support for the first pathway of the proposed model, as social anxiety predicted depressive symptoms over time but not vice versa—thus supporting the majority of the current literature [4]. Cybervictimization did not significantly mediate the links between social anxiety and depressive symptoms, however. Instead, Time-2 depressive symptoms and social anxiety, respectively, mediated the links between Time-1 social anxiety and Time-3 depressive symptoms for both boys and girls. Thus, Time 1 internalizing variables predicted Time 3 variables via Time 2 variables. These findings support the notion that initial levels of depressive symptoms and social anxiety at Time 1 predict changes in depressive symptoms and social anxiety at Time 3, respectively, through subsequent levels of depressive symptoms and social anxiety at Time 2. One gender difference on these links emerged, as depressive symptoms predicted an increase in cybervictimization for boys, but not for girls. In sum, our results indicate that social anxiety is a temporal precursor to depressive symptoms, whereas depressive symptoms predict boys’ increased cybervictimization over time.

Despite Time-1 depressive symptoms predicting Time-2 cybervictimization for boys but not for girls, there were no differences in length or frequency of chatting nor mean levels of cybervictimization between genders. This is different compared to literature showing that girls report higher levels of cybervictimization [29]. Other studies have shown that cybervictimized boys report higher levels of somatic symptoms compared to girls in a study of Italian early adolescents [19]. Nevertheless, that study employed cross-sectional data, making it difficult to compare to the current study framework. Another study found that cybervictimization predicted depressive symptoms instead [25]. The difference between those findings and the current ones could be attributed to a different longitudinal methodology employed, with only 6 weeks between the time points and with an ethnically diverse US sample. In addition, our measure of cybervictimization refers to actively chatting with others, indicating an active online social context rather than random online harassment, and the questions regard for example being harassed for no apparent reason, being abused or insulted, and being teased or made fun of during chat sessions. Compared to the previous literature focusing on offline victimization, then, the current findings imply that internalizing psychopathology has distinct consequences in terms of cybervictimization for boys in particular. Displaying weakness in any form might be particularly detrimental for boys during early adolescence, especially in chat rooms where such displays are likely open to others. Perhaps boys’ demonstration of depressive symptoms left them more vulnerable to cyberbullying compared to girls, whose depressive symptoms might be more accepted bearing in mind current gender stereotypes. In addition, even though we do not know who the adolescents were chatting with, there is a high likelihood that they knew their cyberbullies offline as well, as adolescents who are victimized at school tend to be victimized by the same schoolmates in online settings [15]—and this might be particularly true for boys. Needless to say, these results are worrisome for boys in particular.

To our knowledge, this is one of the few studies to test the multiple pathways model directly, in order to examine co-morbidity between social anxiety and depressive symptoms in a non-clinical sample. In one other study testing the same model, the authors found that Time-1 peer victimization predicted social anxiety at Time 2, which subsequently predicted higher levels of social anxiety at Time 3 (with approximmately 9 months between the time points; 14). This study supported pathway 2 in the proposed model, as interpersonal stressors, familial emotional maltreatment, and peer victimization were linked to the co-occurrence of social anxiety and depressive symptoms—regardless of gender. Nevertheless, there are some methodological differences between the aforementioned study and the current one. First, the participants in the study conducted by Hamilton and colleagues [14] were slightly younger adolescents from the US, and 51% were African–American youths. In addition, nearly half of the sample were eligible for subsidized lunch, which implies adolescents with lower SES. In addition to peer victimization, the authors also examined familial and emotional maltreatment such as abuse and neglect, and stressful life events as potential mediators between social anxiety and depressive symptoms. Nonetheless, the authors did not conduct a fully cross-lagged path model as measures of interpersonal stressors were only available at Time 2. Importantly, they also did not examine cybervictimization in particular. Finally, there was a low level of stability for the measure of social anxiety from Time 2 to Time 3. Thus, the differences between this prior study and the current study sample and measures might help explain why the authors did not find support for pathway 1. In contrast, the current study included all measures at all time points—thus controlling for the effects of the mediators across time. In addition, our study is in line with many others in the literature supporting pathway 1 [e.g., 4, 11].

The current study has several limitations. First, we only used self-reports for all measures in the study, which are always subject to bias and not necessarily reflective of actual behavioral expressions. Social anxiety, however, might not for instance always be obvious to others, and individuals themselves seem to be the best judges of their own social fears [49]. Similar reasoning might be applied to depressive symptoms as well. In addition, we used a cybervictimization measure that reflects being victimized while chatting with others online. Relational aggression as well as more random online harassment were not examined, but these might be equally important—albeit in other ways. In addition, distinguishing between peer- and self-reports of cybervictimization would have been preferable, as peer perceptions of victimization have been linked to peer rejection, whereas self-ratings of victimization have been associated with various adjustment problems such as self-esteem, anxiety, loneliness and depression [50, 51]. Also, the data were collected between 2010 and 2012. Even though this is not that long ago, relatively speaking, a lot has happened in terms of cybervictimization with the emergence of smart phones and tablets. Future studies should therefore probe more current varieties of cybervictimization. Also, several of the participants were recruited by friends who were already involved in the study, which might have impacted the role of social anxiety and possibly other constructs in the study. Nevertheless, there were no significant differences between the initial participants and the snowballed sample on any of the study variables. We also had some missingness in our data, which we have dealt with by means of estimation, but this is a limitation shared by many other studies collecting longitudinal data from young people. Finally, we used data with approximately 8 months between the time lags, which might not be the most appropriate intervals in terms of detecting associations between the constructs used in the study, as the changes between the variables might happen either at a faster or a slower pace than the 8 months measurement points. Despite its limitations, nevertheless, the current study has several strengths. First, we used a longitudinal sample of early adolescents followed for three time points, thus allowing for testing temporal sequences between social anxiety and depressive symptoms, as well as the potential mediating role of cybervictimization. Second, our sample was also representative for Swedish early adolescents at the time of the data collection. Third, we tested for all pathways in one single model in the proposed multiple pathways model. Thus, the current study provides a distinctive insight into how co-morbidity between social anxiety and depressive symptoms in early adolescence might develop.

Early adolescence is characteristically the time of onset for both non-clinical social anxiety [1, 2] and depressive symptoms [3, 4], which tend to increase during this time period [5] and show high levels of co-morbidity throughout adolescence [2]. Because early adolescent social anxiety and depressive symptoms likely precede social anxiety disorder as well as major depressive disorder later on in life [6, 7], understanding more about the co-morbidity between them is therefore of consequence if future problems are to be prevented. Early adolescence also signifies a distinct change in social dynamics that seems to foster victimization by peers [15]. Despite some arguments that cyberbullying might be over-rated as a phenomenon [see e.g., 48], studies on cybervictimization indicate that it is linked to particular emotional difficulties regardless of offline victimization [19–22]. In fact, cybervictimization can be more difficult to escape compared to for example being bullied in-school, as online bullies can follow a person everywhere via smart phones and other devices [19]. There are several important implications from the current study. First, the findings suggest that early adolescent social anxiety has an impact on depressive symptoms over time, but not the other way around—supporting an array of other studies in the area [4]. Nevertheless, this process was not indirectly affected via cybervictimization. Depressive symptoms did predict an increase in cybervictimization for boys, however, which is a finding requiring further probing in the future. In order to address the comorbidity between social anxiety and depressive symptoms, prospective efforts should be targeted to address early adolescent social anxiety in particular. Our findings also indicate that depressive symptoms predicted subsequent cybervictimization for boys—making it essential to remain vigilant about these processes for them in particular.

## Summary

The current study focuses on the temporal sequence of social anxiety and depressive symptoms, and whether interpersonal stressors such as cybervictimization mediate these links. We used a longitudinal sample of 501 early adolescents aged between 13 and 15 followed at three time points. We collected information on symptoms of social anxiety, depression, and perceptions of cyberbullying. We examined longitudinal links between our main study variables (whether social anxiety predicts depressive symptoms, whether depressive symptoms predict social anxiety, or both), as well as to what extent cybervictimization mediated and gender moderated these links. Our results show that social anxiety was a predictor of depressive symptoms over time, but not the other way around. We also found that Time-1 depressive symptoms predicted Time-2 cybervictimization, but only for boys and not for girls. Thus, boys fared worse in terms of cybervictimization after reporting depressive symptoms compared to girls. Cybervictimization did not mediate the links between social anxiety and depressive symptoms; however, Time-2 social anxiety was a significant mediator between Time-1 social anxiety and Time-3 depressive symptoms, whereas Time-2 depressive symptoms were a significant mediator between Time-1 social anxiety and Time-3 depressive symptoms. In conclusion, social anxiety is a strong precursor of depressive symptoms over time but not vice versa—irrespective of interpersonal stressors such as cybervictimization. The results from the current study point to the importance of targeting efforts to address early adolescent social anxiety, as well as being observant about boys’ internalizing in particular.
